# Nutritional Therapy in Cancer Patients Receiving Chemoradiotherapy: Should We Need Stronger Recommendations to Act for Improving Outcomes?

**DOI:** 10.7150/jca.31611

**Published:** 2019-07-10

**Authors:** Paolo Cotogni, Paolo Pedrazzoli, Elisabeth De Waele, Giuseppe Aprile, Gabriella Farina, Silvia Stragliotto, Francesco De Lorenzo, Riccardo Caccialanza

**Affiliations:** 1 Pain Management and Palliative Care, Department of Anesthesia, Intensive Care and Emergency, Molinette Hospital, University of Turin, Turin, Italy; 2 Medical Oncology Unit, Fondazione IRCCS Policlinico San Matteo and Department of Internal Medicine, University of Pavia, Pavia, Italy; 3 Intensive Care Unit and Department of Nutrition, UZ Brussel, Vrije Universiteit Brussel (VUB), Brussels, Belgium; 4 Department of Oncology, San Bortolo General Hospital, Vicenza, Italy; 5 Department of Oncology, ASST Fatebenefratelli Sacco, Milan, Italy; 6 Medical Oncology 1, Veneto Institute of Oncology-IRCCS, Padova, Italy; 7 FAVO Italian Federation of Volunteer-based Cancer Organizations, Rome, Italy; 8 Clinical Nutrition and Dietetics Unit, Fondazione IRCCS Policlinico San Matteo, Pavia, Italy.

**Keywords:** malnutrition, dose-limiting toxicity, nutritional support, artificial nutrition, guidelines

## Abstract

One of the challenges during chemotherapy and radiotherapy is to complete the planned cycles and doses without dose-limiting toxicity. Growing evidence clearly demonstrates the relationship between dose-limiting toxicity and low muscle mass. Moreover, malnutrition leads to low performance status, impaired quality of life, unplanned hospital admissions, and reduced survival.

In the past, the lack of clear and authoritative recommendations and guidelines has meant that oncologists have not always fully appreciated the importance of nutritional therapy in patients receiving anticancer treatments. Therefore, collaboration between oncologists and clinical nutrition specialists needs to be urgently improved.

Recent guidelines from scientific societies and practical recommendations by inter-society consensus documents can be summarized as follows: 1) timely nutritional therapy should be carefully considered if patients undergoing anticancer treatments are malnourished or at risk of malnutrition due to inadequate oral intake; 2) if oral intake is inadequate despite counseling and oral nutritional supplements, supplemental enteral nutrition or, if this is not sufficient or feasible, parenteral nutrition should be considered; 3) home artificial nutrition should be prescribed and regularly monitored using defined protocols developed between oncologists and clinical nutrition specialists; 4) appropriate nutritional management in the context of simultaneous care should become a guaranteed right for all patients with cancer.

The purpose of this review is to provide oncologists with an overview of the aims and current evidence about nutrition in oncology, together with updated practical and concise recommendations on the application of nutritional therapy in cancer patients receiving chemoradiotherapy.

## Introduction

In 1998, Andreyev et al. first reported that cancer patients with weight loss had worse outcomes when undergoing chemotherapy (CT) 1. The authors demonstrated that the poorer outcome in these patients appeared to occur because they received significantly less CT and developed more frequent and more severe toxicity rather than any specifically reduced responsiveness of tumors to chemotherapeutic regimens. In 2004, Ross et al. found that lung cancer patients who had lost weight failed to complete at least 3 cycles of CT more frequently than did those with stable weight. In addition, these patients had more symptoms at presentation, a trend towards reduced symptomatic benefit from CT, and a significantly increased risk of death 2.

In the last 10 years, many studies assessing outcomes in cancer patients undergoing CT have clearly shown that lean body mass loss is an independent risk factor for dose-limiting toxicity, hospitalization, and survival. Moreover, patients who are malnourished at the start of treatment experience a further nutritional decline during CT 3-10. Likewise, low muscle mass has been shown to be a risk factor for increased toxicity of targeted therapies and multikinase inhibitors, with weight loss being a common adverse effect of these treatments 11.

Despite all these reports, a 1-day prevalence survey carried out in 154 French hospital wards on 1903 cancer patients showed that a high rate (42%) of malnourished patients were still not receiving nutritional therapy 12.

## Barriers to effective nutritional practice

Although it is recognized that weight loss is not simply an irreversible marker of cancer patients with expected negative outcomes, the literature suggests that in patients receiving CT the benefit of nutritional support, either enteral or parenteral, has not been adequately considered by oncologists.

In 2006, a survey carried out in the United Kingdom suggested that specialist oncological trainees lack the ability to identify factors that place cancer patients at risk of malnutrition 13. Two-thirds of trainees rated nutritional status as very important in patient's morbidity and quality of life (QoL). However, these physicians were least likely to agree that nutritional intervention would reduce mortality in cancer patient with a severe weight loss. The study concluded that the 3 most important barriers to nutritional interventions reported by the trainees were lack of clear guidelines (69%), lack of knowledge or training in this area (60%), and time constraints preventing referral for, or direct nutritional interventions (56%).

Similarly, in 2016 another survey reported the lack of awareness and consideration of nutritional issues among Italian oncologists 14. Although both malnutrition and nutritional therapy seemed to be perceived by the responders as important factors for the efficacy of oncologic treatments, it nevertheless appeared that nutritional support management may well be inadequate. The survey showed a lack of structured collaboration between oncologists and clinical nutrition specialists as well as a need for practical recommendations on nutritional therapy in oncology patients.

In the same year, Martin et al. carried out a qualitative study through interviews at 5 European centers (France, Italy, the Netherlands, Scotland, and Sweden) investigating the barriers to the implementation of nutritional care in patients with head and neck or esophageal cancers 15. Five factors acting as barriers to, or enablers in, nutritional care were identified: (a) evidence base for the benefit of nutritional interventions; (b) implementation processes for nutritional care (assessment, intervention, and follow-up); (c) provider characteristics (awareness, knowledge and training, professional roles, motivation and outcomes expectancy); (d) site factors (resources for implementation and hospital structure); (e) patient characteristics (preferences and motivation).

## Recommendations and guidelines on nutrition in cancer patients

In the past, there has been insufficient appreciation of the clinical benefits of nutritional therapy during anticancer treatments, due to a lack of clear, authoritative, and shared recommendations or guidelines on the issue. Collaboration between oncologists and clinical nutrition specialists has been poor with oncologists struggling to identify patients at risk of malnutrition due to deficiencies in training and lack of time.

The development of standardized nutritional care pathways is complex and requires nutritional care to be fully integrated as part of the multimodal care process in cancer patients. Improving the nutritional care process requires the involvement of several healthcare providers across different disciplines (surgery, medical oncology, radiotherapy, and clinical nutrition), together with an explanation of roles and responsibilities involved in the provision of nutrition throughout the continuum of care. This message has to be disseminated both to professionals 16 as well as to cancer patients and their families 17.

For this purpose, a working group was established, with members coming from the Italian Association of Medical Oncology (AIOM), the Italian Society of Artificial Nutrition and Metabolism (SINPE), and the Italian Federation of Volunteer based Cancer Organizations (FAVO), with the objective of initiating a structured collaborative project named “Integrating Nutritional Therapy in Oncology” (INTO) 18.

This working group in 2016 released an inter-society consensus document detailing appropriate nutritional support in cancer patients 19. The main practical recommendations may be summarized as follows: (a) nutritional screening should be performed using validated tools, at diagnosis and at regular time points in patients at risk of malnutrition; (b) patients at risk of malnutrition should be promptly referred for comprehensive nutritional assessment and support to personnel with documented skills in clinical nutrition, specifically for cancer patients; (c) nutritional support should comprise dietary counseling with the possible use of oral nutritional supplements (ONS) and/or enteral nutrition (EN), total or supplemental parenteral nutrition (PN) according to spontaneous food intake, tolerance, and effectiveness; (d) “alternative hypocaloric anticancer diets” (e.g. macrobiotic or vegan diets) are not recommended; (e) nutritional support may be integrated into palliative care programs, according to individual-based evaluations, QoL implications, life expectancy and patients' awareness; (f) home artificial nutrition should be prescribed and regularly monitored using defined protocols shared between oncologists and clinical nutrition specialists.

Recently, the working group also elaborated a “Cancer Patients' Bill of Rights for appropriate and prompt Nutritional Support” 20. The members of the working group are convinced that appropriate nutritional management in the context of simultaneous care should become a guaranteed right for all patients with cancer. One of the aims of this document is to make cancer patients aware of their rights with regard to nutritional care as well as alerting public opinion and healthcare institutions to the neglected problem of malnutrition in oncology.

In 2016, the European Society for Clinical Nutrition and Metabolism (ESPEN) released a new version of the guidelines on nutrition in cancer patients 21. The main recommendations are: (a) food intake is considered inadequate if a patient has been unable to eat for more than a week, or if the estimated energy intake is less than 60% of requirement for more than 1-2 weeks (Strength of recommendation: Strong; Level of evidence: Moderate); (b) muscle protein depletion is a hallmark of cancer cachexia, severely impinging QoL and negatively impacting physical function and treatment tolerance (Strength of recommendation: Strong; Level of evidence: Moderate); (c) nutritional intake, weight change, and body mass index (BMI) should be regularly monitored following cancer diagnosis and repeated depending on the stability of the clinical situation (Strength of recommendation: Strong; Level of evidence: Very low); (d) in patients with chronic insufficient dietary intake and/or uncontrollable malabsorption, home artificial nutrition (either enteral or parenteral) should be used in suitable patients (Strength of recommendation: Strong; Level of evidence: Low); (e) specifically in patients undergoing anticancer treatments, if oral food intake is inadequate despite counseling and ONS, supplemental EN or, if this is not sufficient or possible, PN should be implemented (Strength of recommendation: Strong; Level of evidence: Very low).

A crucial issue is also the timing of nutritional interventions. A window of anabolic potential seems to exist when survival is greater than 90 days, creating a chance for nutritional intervention to stop or reverse cachexia 22. Artificial nutrition can maintain or improve nutritional status in cancer patients, but only if depletion of lean body mass is not extreme. Rather than attempting to reverse severe weight losses in advanced stages of cancer disease, nutritional therapy would be more successful if started at the initial phases.

Similarly to pain management and palliative care 23, the early integration of nutritional care into oncology may improve perceived QoL, and reduce both patient and caregiver distress due to nutrition-impact symptoms like appetite loss, nausea, taste changes, dysphagia, early satiety, eating-related abdominal pain, diarrhea, and constipation. Early referral to a clinical nutrition expert may also enhance the patient's ability to cope with psychosocial distress associated with inadequate food intake and weight loss, and, above all, it allows tailoring nutritional therapy to patient's individual nutritional needs, expectations, preferences, and wishes.

## Evidence supporting nutritional interventions

There is agreement that unconditional artificial nutrition in all patients undergoing anticancer treatments is not indicated, but also that nutritional therapy should be carefully considered if these patients are malnourished or at risk due to an inadequate food intake 21. Less is known on whether nutritional support (i.e. artificial nutrition) improves survival of cancer patients with advanced disease, beyond its role in ameliorating anthropometric measures and domains of QoL. Moreover, survival results were obtained by few studies not powered to assess mortality.

Evidence of the benefits of nutrition in cancer patients from clinical trials is extremely necessary. Indeed, the collection of data supporting nutritional interventions could be one of the main actions that overcome the barriers hampering the provision of nutrition in these patients 19. Actually, the lack of implementation of nutritional support in clinical practice could be secondary to the low level of the available evidence rather than to the inappropriate wording of the recommendations.

Of course, in long-term studies involving patients with chronic insufficient oral intake it would have been ethically unacceptable to have the control arm not receiving any nutritional support. So, randomized controlled trials (RCTs) on the effectiveness of EN or PN supplementation are ruled out 24. Moreover, in medical oncology it could be difficult to complete large prospective randomized trials due to the competition with studies sponsored by big pharma companies and including naïve cancer patients.

Nowadays, there is a wide agreement that early consultation with a professional (physician and dietician) with documented skills in clinical nutrition, specifically for oncology patients, is beneficial for cancer patients receiving anticancer treatments 19, 21, 25-27 as well as for those in the advanced stages of disease 22. However, there are few clinical studies investigating the effects of nutritional support in cancer patients undergoing CT or radiotherapy (RT).

### Dietary counseling and oral nutritional supplements

Dietary counseling, including the use of ONS, should be the first-step toward increasing the oral energy and protein intake with the aim of improving clinical outcomes 19, 21.

In patients undergoing adjuvant RT there is good evidence that dietary counseling improves oral intake, body weight, and some aspects of QoL as well as helping to reduce the incidence and severity of toxicity, thereby avoiding treatment interruptions 28-31. A systematic review and meta-analysis by Baldwin et al. examined the evidence for an effect of dietary intervention (nutritional counseling, ONS, or both) in 1414 cancer patients receiving both chemotherapy and radiotherapy given as adjuvant, neoadjuvant, or primary treatment who were malnourished or were at risk of malnutrition 32. The authors found that the global QoL, emotional functioning, dyspnea, and loss of appetite scales, but not mortality, were significantly improved in patients undergoing oral nutritional interventions.

Despite the small number of patients enrolled in the RCT (i.e. 37 vs. 74), Ravasco et al. demonstrated an improved survival (median follow-up of 6.5 years) in colorectal cancer patients who received early individualized dietary counseling during RT 33.

Cereda et al. performed a RCT in head and neck cancer patients undergoing RT or RT plus systemic treatment and receiving nutritional counseling 34. In this appropriately sized study, the additional provision of ONS resulted in better weight maintenance, increased protein-calorie intake, improved QoL, and was associated with better anticancer treatment tolerance.

### Enteral nutrition

In case of oral nutrition remains inadequate despite counseling and ONS, and in presence of normal gut function, total or supplemental EN should be considered 19, 21.

Prospective and retrospective observational trials in patients with obstructing head and neck cancers undergoing RT or CT demonstrated that EN (better if early) compared with oral feeding reduces weight loss, frequency, and duration of treatment interruptions and the rate of hospital admissions 35-37.

Miyata et al. carried out a RCT in non-malnourished patients with esophageal cancer receiving neoadjuvant CT and supplemental artificial nutrition (600 kcal/day), and showed that EN during CT reduces CT-related adverse hematological events 38. In 347 stage IV gastric cancer patients at high nutrition risk and receiving palliative CT, nutritional support was associated with a survival benefit 39.

A cohort trial in colorectal cancer patients undergoing CT reported a longer survival (19.1 vs. 12.4 months) in 315 patients receiving counseling, ONS, and megestrol acetate compared with 313 patients without nutritional support 40. A randomized pilot trial in cachectic cancer patients compared standard nutritional treatment with an individualized nutritional intervention program, which was escalated from counseling to ONS, EN, and PN, as required, to avoid a caloric deficit 41. This individualized nutritional intervention program was associated with improved body weight and reduced unexpected hospital stay as well as survival, but the study was not powered to assess mortality. A multicenter RCT in post-surgical malnourished patients with upper gastrointestinal cancers showed that patients on home EN had a higher chance of completing the planned CT compared with those receiving nutritional counseling only (48% vs. 34%) 42.

### Parenteral nutrition

When EN is not feasible, insufficient or contraindicated, supplemental or total PN ensures that cancer patients receive adequate nutritional therapy 19, 21. However, the use of PN in cancer patients undergoing chemoradiotherapy has been debated for years because many physicians were concerned about the risks (i.e. infections and venous thrombosis) potentially associated with the use of central venous catheters.

In a prospective study of over 51000 catheter days in cancer patients (45% receiving chemoradiotherapy) on home parenteral nutrition (HPN), the incidence of catheter-related bloodstream infections was 0.6/1000 HPN-days 43. Thus, if carefully managed, HPN can be safely provided to cancer patients undergoing anticancer treatments without expecting a significant incidence of catheter-related complications, both infections and thrombosis 43, 44.

For many years, the role of HPN in advanced incurable cancer patients has been a controversial topic 45-47. Actually, advanced cancer patients may have a life expectancy of several months to several years. In these patients, malnutrition impairs performance status, QoL, tolerance to anticancer treatments, and survival 21. For ethical reasons the benefit of HPN has not been investigated in RCTs. However, over the last ten years several prospective and retrospective observational studies in advanced cancer patients with inadequate oral intake receiving chemoradiotherapy showed that HPN improves QoL 48-54 and prolongs survival 46, 55-58.

In particular, in a prospective study in 111 advanced cancer patients on HPN, those receiving anticancer treatments (65%) showed higher QoL scores than patients with no treatment 53. Therefore, it is possible that in these patients the benefits of HPN outweighed the adverse effects of anticancer treatments. Conversely, a prospective study in 158 patients with end-stage cancer showed that palliative CT was not associated with survival, did not improve QoL for patients with moderate or poor performance status and actually worsened QoL of patients with good performance status 59. Overall, these data indicate a need to consider the benefits of supplemental HPN in combination with chemoradiotherapy in the palliative phase.

The indication for HPN in incurable patients who are unable to eat mainly for malignant intestinal obstructions is extremely controversial. However, even in aphagic patients with advanced cancer receiving no anticancer treatment, HPN was shown to improve QoL 60, 61 and survival 62-67. As a matter of fact, there are observational studies showing HPN increased survival by many months and even years in incurable advanced cancer patients, especially in those with initially preserved performance status. Indeed, patients who have low Karnofsky performance status (KPS) (≤ 50) or poor Eastern Cooperative Oncology Group (ECOG) score (≥ 3) are less likely to benefit from HPN 56, 62, 65, 67, 68.

In a Swedish observational study of the prevalence and use of artificial nutrition in palliative patients, 22% of inpatients and 11% of those in home care services received artificial nutrition, with PN being most common 69.

The start of HPN should be carefully considered together with the patient, taking into account his/her wishes, expected benefits on QoL, and prognosis as well as the likely burden for patients and caregivers 21. Prognosis is obviously the most important conditioning issue. The European and American societies for clinical nutrition have recommended considering PN if expected survival of cancer patient is more than 1-3 months 21, 70. Actually, predicting survival in incurable cancer patients is not easy and should include clinical judgement and use of validated scoring systems 68, 71.

Several studies have highlighted the importance of assessing body composition in cancer patients 72, 73. Bioelectrical impedance vectorial analysis (BIVA) is a non-invasive, validated method to assess body composition, which offers an efficacious way to track changes over time and in different clinical settings 74. Particularly, some output measures of BIVA (i.e. phase angle and fat free mass) are correlated with nutritional status 4, 75 and energy intake 76, and are independent predictors of QoL and survival in cancer patients 77. Therefore, the assessment of body composition by BIVA should be integrated in the nutritional assessment of cancer patients 19.

Two recent prospective studies have investigated the effects of PN in cancer patients receiving chemoradiotherapy using also BIVA 78, 79. Early 7-day supplemental PN resulted in significantly improved phase angle, handgrip strength, and serum prealbumin levels in 118 hypophagic and hospitalized cancer patients at nutritional risk 78.

After 90 days of HPN, malnourished cancer outpatients experienced significantly improved nutritional status (Patient-Generated Subjective Global Assessment and BMI), performance status (KPS), prognostic score (modified Glasgow prognostic score), and some BIVA measures 79. Moreover, reactance, resistance, and phase angle were significantly associated with survival at T0, T1, and T2, respectively 79.

### Low muscle mass, fasting, and response to cancer therapy

Fasting periods have emerged as promising strategies to target clinical parameters that constitute the foundation for metabolic syndrome, cardiovascular disease, cancer, and neurodegenerative diseases. Although promising, these approaches are still experimental in nature and should not be initiated without medical supervision 80.

The evidence provided by human studies regarding the benefit of fasting and calorie restriction before and during CT is still very limited 16, 81. Several trials are currently underway to determine the potential for short-term fasting in reducing the side effects and enhancing the efficacy of CT, but the results have not yet been published 82.

A recent review reported that several recent studies in cancer outpatient populations found that higher amounts of muscle mass were associated with improved survival 83. Finally, a very recent authoritative editorial concludes that there is growing observational evidence that measures of body composition are associated with numerous outcomes in patients with cancer; specifically, these measures can be used to identify patients who are most likely to experience adverse events and toxicities from CT 84.

## Conclusions

One of the challenges during chemotherapy and radiotherapy is to complete the planned cycles and doses without dose-limiting toxicity. The relationship between dose-limiting toxicity and muscle mass loss has been clearly recognized. Similarly, malnutrition is a significant determinant of performance status and QoL.

In conclusion, the crucial question is: should we need stronger recommendations to act for improving outcomes? Well, the answer is no, but we certainly need more robust clinical data to convince the entire international Oncology community as soon as possible. Oncologists should be aware that nutritional therapy must be timely considered and prescribed, when indicated, to all malnourished or at-risk-of-malnutrition cancer patients receiving chemoradiotherapy, as any delay in nourishing these patients might compromise the potential benefits of nutritional therapy.

## Abbreviations

AIOM: Italian Association of Medical Oncology
BIA: bioelectrical impedance analysis
BMI: body mass index
CT: chemotherapy
ECOG: Eastern Cooperative Oncology Group
EN: enteral nutrition
ESPEN: European Society for Clinical Nutrition and Metabolism
FAVO: Italian Federation of Volunteer based Cancer Organizations
HPN: home parenteral nutrition
INTO: Integrating Nutritional Therapy in Oncology
KPS: Karnofsky performance status
ONS: oral nutritional supplements
PN: parenteral nutrition
QoL: quality of life
RCT: randomized controlled trial
RT: radiotherapy
SINPE: Italian Society of Artificial Nutrition and Metabolism
